# Human immunodeficiency virus infection acquired through a traditional healer’s ritual: a case report

**DOI:** 10.1186/s13256-017-1458-1

**Published:** 2017-10-26

**Authors:** Pedro Pallangyo, Paulina Nicholaus, Henry Mayala, Andrew Kabeho, Anna Nkinda, Mohamed Janabi

**Affiliations:** Department of Cardiovascular Medicine, Jakaya Kikwete Cardiac Institute, P.O Box 65141, Dar es Salaam, Tanzania

**Keywords:** AIDS, Cultural norms, HIV infection, HIV transmission, Sub-Saharan Africa, Traditional healer, Unsterilized instruments

## Abstract

**Background:**

Globally, over 36 million people were infected with human immunodeficiency virus by the end of 2015. The Sub-Saharan African region home to less than one-fifth of the global population disproportionately harbors over two-thirds of the total infections and related deaths. Residents of Sub-Saharan Africa continue to face limited access to allopathic medicine and it is estimated that over 80% of primary health care needs in the region are met through traditional healing practices. It is known that some of these practices are performed in groups and the use of unsterilized instruments is common thus potentiating the transmission of human immunodeficiency virus.

**Case presentation:**

A 29-year-old business woman of African origin residing in rural Tanzania presented at a screening event to confirm her human immunodeficiency virus status. Her past medical history was unremarkable and so were two past pregnancies. As per the antenatal clinic card for the second pregnancy, her human immunodeficiency virus serostatus was negative. She reported that she had been taken to a traditional healer to take an oath of remaining faithful during her husband’s absence. The oath involved cutting of the healer’s skin followed by hers using the same instrument. Approximately 4 months following this traditional ritual she developed a febrile illness accompanied by enlarged lymph nodes of her neck. She was investigated for malaria, typhoid fever, and urinary tract infection which were negative but she tested positive for human immunodeficiency virus. Owing to her disbelief regarding the human immunodeficiency virus status, she went to three other care and treatment clinics and the results remained similar. She denied any history of transfusion or extramarital affairs. She tested positive at the screening event and enzyme-linked immunosorbent assay for human immunodeficiency virus performed at our institution was reactive. Tenofovir, lamivudine, and efavirenz antiretroviral combination was initiated.

**Conclusions:**

Persistence of cultural norms involving exposure of bodily fluids and use of unsterilized instruments especially in the developing world remains a viable source of human immunodeficiency virus transmission especially in rural areas.

## Background

The human immunodeficiency virus (HIV) pandemic remains a major challenge to global health, economy, and development. Owing to the survival benefits of antiretroviral therapy, the present global trends demonstrate an overall rise in HIV prevalence. The Sub-Saharan Africa region, which carries a disproportionate burden (>70%) of the global HIV infections and related deaths, remains the hardest hit region [1]. For several decades the epidemiological differences among cases of acquired immunodeficiency syndrome (AIDS) in Africa compared with the Western world have raised several speculations regarding potential unique risk factors for transmission in Africa. Such factors include cultural practices resulting in blood exposure with or without the use of shared instruments: medicinal bloodletting, rituals establishing “blood brotherhood,” ritual and medicinal enemas, ritual scarification, male circumcision, female genital mutilation, and genital tattooing [2].

Residents of Sub-Saharan Africa continue to face limited access to allopathic medicine and it is estimated that over 80% of health care needs in the region are met through traditional healing practices [3]. In addition, Sub-Saharan Africa and many other resource-limited nations have more traditional healers than biomedical practitioners [4]. For instance in Tanzania the ratio of traditional practitioners to general population is 1:400 compared to 1:33,000 of medical doctors to population [4]. In addition, knowledge of HIV/AIDS among traditional healers in developing nations is arguably poor. In a local study by Uiso *et al*. for example, over 60% of traditional healers claimed to be treating patients with HIV/AIDS but less than a quarter had good knowledge of the pandemic [5]. Furthermore, 10% of the respondents in the same study claimed to have cured patients with HIV/AIDS [6]. Although the risk of acquiring HIV infection from the use of unsterile instruments is relatively low (0.7%), several practices of traditional healers including scarification, circumcision, and enemas hold a potential for spreading HIV/AIDS [2, 7–9]. We report a case of HIV infection acquired through a traditional healer’s ritual in a 29-year-old woman of African descent.

## Case presentation

A 29-year-old business woman of African descent from Kongwa district, Dodoma, Tanzania, presented at a non-communicable disease screening event organized by the Ministry of Health in February 2017. She had an unremarkable past medical history. She had had two uneventful pregnancies and among other things she tested negative for HIV in both pregnancies. She is of a Christian faith, however through her husband she has visited traditional healers a couple of times.

The purpose of coming to the screening event was to confirm her HIV status and she was accompanied by her husband. She reported a febrile illness accompanied by painful enlarged lymph nodes of her neck 3 weeks prior. She was then investigated for malaria, urinary tract infection, and typhoid which revealed negative results. In addition, she was counseled and tested for HIV which was reactive. Before this index visit, she had gone to three other care and treatment clinics (CTC) because of her disbelief regarding her HIV status; the serostatus results remained unswerving. A rapid HIV test (Determine and SD Bioline) conducted at the screening grounds was reactive for her but her husband tested negative.

She denied ever being blood transfused or having extramarital affairs. However, approximately 4 months ago she reported being taken to a traditional healer by her husband to take an oath. One of her husband’s professional roles requires him to leave home for a couple of days. To ensure that during his absence his wife avoids extramarital affairs, he takes her to a traditional healer to take an oath of remaining faithful. The oath apart from a verbal declaration involved the healer cutting himself and then cutting our patient using the same blade.

We referred her to our facility for further investigations and management. She had stable vital signs (blood pressure 114/71 mmHg, pulse rate 77 beats/minute, respiratory rate 14 breaths/minute, and temperature 37.1 °C) but her body mass index (BMI) fell in the obese range (36 kg/m^2^). Her full blood count, electrolytes, and renal, liver, and thyroid functions were unremarkable. Electrocardiogram (ECG) and echocardiography (ECHO) revealed essentially normal cardiac functions. We performed enzyme-linked immunosorbent assay (ELISA) for HIV for our patient and her husband; she was reactive while her husband was not. Sputum for acid-fast bacilli (AFB) was negative and she had a CD4 count of 316 cells/mL. Furthermore, Venereal Disease Research Laboratory (VDRL) test and hepatitis B and C serologies were negative. Her lipid profile revealed elevated total cholesterol (7.2 mmol/L) and low density lipoproteins (5.6 mmol/L) and triglycerides (3.1 mmol/L). She was further screened for depression using a Patient Health Questionnaire (PHQ-9) which revealed moderate depressive symptoms. Based on the history and laboratory findings, we were convinced that she was a low-risk patient and the most probable source of infection was through the unsterile instrument used during a traditional healer’s ritual.

We further referred her to a CTC within our institution and she was started on a tenofovir, lamivudine, and efavirenz antiretroviral combination. In addition, multivitamins and atorvastatin 40 mg once daily were prescribed. Moreover, we consulted a psychologist for psychoanalysis, psychotherapy, and management of depression.

## Discussion

Globally, over 36 million people were affected by HIV/AIDS by the end of 2015 [10]. It is estimated that approximately 40% of HIV-infected individuals are unaware of their seropositive status; a majority of these reside in resource-limited settings [11]. The Sub-Saharan African region which harbors less than one-fifth of the global population was home to over 25 million people living with HIV/AIDS in 2015 [10]. Moreover, 46% of the new HIV infections in 2015 transpired in Eastern and Southern Africa [10]. While a cure for HIV is yet to be found, prevention remains fundamental in the control of the pandemic.

Since its existence, HIV/AIDS and culture have been thought to have an inextricable link. Regardless of the lack of empirical evidence, such speculations regarding cultural norms as a cofactor in the spread of HIV/AIDS especially in Sub-Saharan Africa have persisted. Traditional surgical practices especially those performed in groups have been documented as potential methods for HIV spread [2, 12, 13]. Such practices including scarification, male circumcision, female genital mutilation, genital tattooing, and ritual venipuncture usually involve the use of unsterilized equipment. For instance it was observed in rural Nigeria that an indigenous medical practitioner performed male circumcision, tattooing, and facial scarification using a single knife by rinsing it in water between patients [13]. Arguably such practices potentially expose patients to bodily fluids of preceding ones as well as those of the traditional healer if they have open wounds.

In the case presented, the woman had delivered approximately 8 months prior and her antenatal clinic card was clear that her HIV serostatus then was negative. Furthermore, her verbal testimony and the fact that she was nursing a baby make it unlikely that she had risky behaviors between delivery and the present time. As the ritual itself involved the traditional healer cutting himself first before cutting the patient using the same blade, the likelihood of our patient being exposed to the healer’s blood (of unknown serostatus) is high. Moreover, the timing between her attendance to the traditional healer and her experiencing symptoms suggestive of acute retroviral syndrome (fever and swollen lymph nodes) makes the hypothesis that the source of infection came from the unsterile instruments of the traditional healer conceivable.

## Conclusions

To conclude, HIV/AIDS remain a significant threat to health and development especially in the developing world. Persistence of cultural norms involving exposure of bodily fluids and use of unsterilized instruments in the resource-limited nations remains a viable source of transmission especially in rural areas. In view of this, traditional healers should continuously receive HIV/AIDS education and other necessary support to make their practices safer.

## Abbreviations

AFB: Acid-fast bacilli
AIDS: Acquired immunodeficiency syndrome
BMI: Body mass index
CTC: Care and treatment clinic
ECG: Electrocardiogram
ECHO: Echocardiography
ELISA: Enzyme-linked immunosorbent assay
HIV: Human immunodeficiency virus
PHQ: Patient Health Questionnaire
VDRL: Venereal Disease Research Laboratory
